# Characterization of age‐associated B cells in early drug‐naïve rheumatoid arthritis patients

**DOI:** 10.1111/imm.13598

**Published:** 2022-11-10

**Authors:** Gemma Vidal‐Pedrola, Najib Naamane, James A. Cameron, Arthur G. Pratt, Andrew L. Mellor, John D. Isaacs, Dagmar Scheel‐Toellner, Amy E. Anderson

**Affiliations:** ^1^ Translational and Clinical Research Institute Newcastle University Newcastle upon Tyne UK; ^2^ Institute for Inflammation and Ageing University of Birmingham Birmingham UK; ^3^ Musculoskeletal Unit Newcastle upon Tyne Hospitals NHS Foundation Trust Newcastle upon Tyne UK; ^4^ Present address: Infectious Diseases Department Yale School of Medicine New Haven Connecticut USA

**Keywords:** age‐associated B cells, chemokine receptors, FcRL family, rheumatoid arthritis, transcriptome

## Abstract

Age‐associated B cells (ABCs) are an immune cell subset linked to autoimmunity, infection and ageing, and whose pathophysiological importance was recently highlighted using single cell synovial tissue profiling. To elucidate their pathophysiological relevance, peripheral blood (PB) ABCs from early rheumatoid arthritis (eRA) patients naïve to disease‐modifying anti‐rheumatic drugs (DMARDs) were compared with their synovial fluid (SF) counterparts, and to PB ABCs from psoriatic arthritis patients and healthy controls. PB and SF B‐cell subsets were phenotyped by multi‐parameter flow cytometry, sorted and subjected to gene expression profiling (NanoString nCounter® Immunology V2 Panel) and functional characterization (stimulated cytokine measurements by immunoassay). PB ABCs of eRA patients, which are transcriptionally distinct from those of control cohorts, express chemokine receptors and adhesion molecules, such as CXCR3, that favour homing to inflammatory sites over lymphoid tissue. These cells are an activated, class‐switched B‐cell subset expressing high levels of HLA‐DR, co‐stimulatory molecules and T‐bet. Their secretion profile includes IL‐12p70 and IL‐23 but low levels of IL‐10. High surface expression of FcRL family members, including FcRL3, furthermore suggests a role for these cells in autoimmunity. Finally, and unlike in the periphery where they are rare, ABCs are the predominant B‐cell subsets in SF. These observations indicate the predilection of ABCs for inflammatory tissue in RA, where their propensity for antigen presentation and pro‐inflammatory phenotype may support autoimmune pathology. Their potential as a therapeutic target therefore warrants further study.

## Graphical abstract

Age‐associated B cells have a distinct transcriptome compared to other B‐cell subsets. These cells express inflammatory‐homing chemokine receptors, as well as FcRL family members that are linked to autoimmune disease. Furthermore, B cells that resemble age‐associated B cells are enriched in rheumatoid arthritis synovial fluid. Age‐associated B cells are a potentially pathogenic cell in rheumatoid arthritis and are a novel therapeutic target that warrant further investigation.

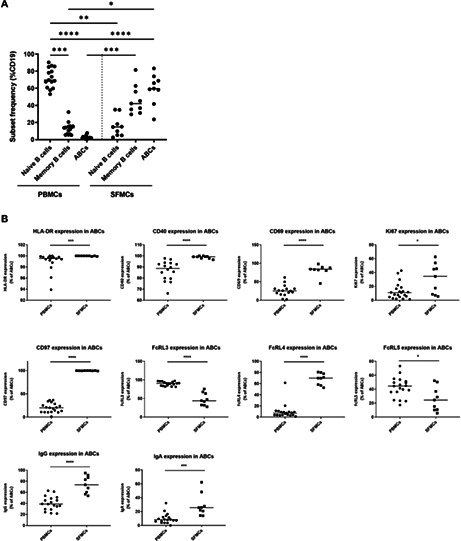

## INTRODUCTION

Rheumatoid arthritis (RA) is a chronic autoimmune disorder of unknown aetiology, which that is characterized by joint inflammation. Many studies focus on the important role of CD4+ T cells; however, the presence of autoantibodies years before onset and the efficacy of B‐cell‐depleting therapies indicate a key role for B cells in RA initiation and progression [1]. Studying disease‐modifying anti‐rheumatic drug (DMARD)‐naïve early RA (eRA) should yield novel insights into pathogenesis that translate into improved therapies and quality of life for patients.

There are several established B‐cell subsets with diverse functionality, including antibody production, antigen presentation to T cells and immune regulation. Investigation of B cells in aged mice identified a subset, named age‐associated B cells (ABCs), expressing the integrins, CD11b and CD11c, but displaying reduced expression of the complement receptor type 2, CD21 [2, 3, 4]. ABCs were also found to be expanded in autoimmune‐prone mice. Human ABCs (CD19^high^CD21^−^CD11c^+^) have been detected in the peripheral blood (PB) of RA, systemic sclerosis and systemic lupus erythematosus (SLE) patients [3]. Other ABC‐like cells, such as CD21^−/low^ B cells and CD20^+^T‐bet^+^CD11c^+^ B cells, are also found in autoimmune patients [5, 6, 7], and are enriched in the synovial fluid (SF) of patients with active RA [8, 9], as well as the synovium [10]. Additionally, we have described FcRL4^+^ SF B cells with an immunophenotype that partially overlaps with ABCs [11, 12].

Human ABCs are reported to have an activated phenotype with high expression of MHC class II and the co‐stimulatory molecules, CD80 and CD86 [3, 7, 13]. Moreover, T‐bet has been described as a specific transcription factor for human ABCs [14, 15]. Further studies demonstrated the potential ability of murine ABCs to express the cytotoxic molecules, perforin and granzyme A, produce autoantibodies, cytokines, and present antigens [3, 16, 17], highlighting a possible role in autoimmunity.

Detailed understanding of ABCs is hampered because these cells are defined in different ways by different investigators, as well as being heterogeneous in surface marker expression and, hence, presumably, function [18]. For this reason, studies characterizing ABCs from different disease states in more detail, focussing on gene expression, phenotype and function should enable better elucidation of their role in health and disease. To this end, we here characterize human ABCs transcriptionally and phenotypically, as well as assessing their functional cytokine profile, in DMARD‐naïve eRA patients, psoriatic arthritis (PsA) patients as a disease control and healthy controls (HCs).

## METHODS

### Patients

Ethical approval for the recruitment of patients was provided by the North East–Newcastle & North Tyneside 2 Research Ethics Committee, REC reference 12/NE/0251. In addition, ethical approval for healthy volunteers was provided in the context of two projects: (1) provided by the County of Durham and Tees Valley Research Ethics Committee, REC reference 12/NE/0121; and (2) obtained from The Animal Welfare and Ethical Review Body (AWERB), Newcastle University, project ID Number: ID 633. Patients referred from primary care with recent onset arthritis who were naïve to DMARDs were recruited from the Newcastle Early Arthritis Clinic, in the Musculoskeletal Unit at the Freeman Hospital, Newcastle upon Tyne. Patients were diagnosed with RA with reference to the 2010 ACR/EULAR classification criteria [19]. These patients are referred to as having eRA due to their recent disease onset. Patients with established RA (estRA) (>1 year duration on treatment) were also recruited from the Musculoskeletal Unit at the Freeman Hospital, Newcastle upon Tyne. All healthy volunteers had no personal history of autoimmunity or other musculoskeletal conditions. All subjects gave written informed consent before inclusion in the study. The demographics and clinical characteristics of all donors are shown in Tables S1–S3.

### PB mononuclear cell isolation

PB mononuclear cells (PBMCs) were isolated from EDTA‐treated blood by density centrifugation on Lymphoprep (Axis‐Shield Diagnostics Ltd.). PBMCs were used immediately for flow cytometry or cell sorting.

### SF mononuclear cell isolation

SF mononuclear cells (SFMCs) were isolated from SF by incubation with heparin (1 U/ml) and hyaluronidase (10 U/ml) for 30 min at 37° celsius before density centrifugation on Lymphoprep. SFMCs were frozen at −80° celsius in FCS containing 10% dimethyl sulfoxide (Sigma‐Aldrich) using a CoolCell to facilitate controlled freezing.

### Flow cytometry––whole blood staining and intracellular staining

Two hundred microliters of EDTA‐treated blood was stained with each antibody panel (see Table S4) at 37° celsius for 30 min before treatment using BD FACS Lysing solution (BD Biosciences) as per the manufacturers' instructions. To assess Ki67 and T‐bet expression, following surface marker staining, cells were permeabilized in Permeabilization Buffer (eBioscience) for 30 min at 4° celsius. To reduce background staining, cells were blocked with 2% mouse serum for 15 min at 4° celsius before adding anti‐Ki‐67 and anti‐T‐bet antibodies (Table S4) for 30 min at 4° celsius. Stained cells were resuspended in FACS buffer (PBS containing 0.5% BSA, 1 mM EDTA and 0.01% sodium azide) and acquired on a BD LSR Fortessa X20 (BD Bioscience). Flow cytometry data were analysed using FlowJo Version 10 (Treestar Inc.). An example gating strategy for phenotypic marker assessment is shown in Figure S1a.

Due to high homology between the FcRL family members, the antibodies used for their detection were validated for specificity to make sure they were not cross‐reactive with other family members (Figure S2). Due to cross‐reactivity with FcRL5, the anti‐FcRL2 clone REA474 was not used for phenotypic analysis. FcRL2 expression was assessed using the anti‐FcRL2 clone B24 (kindly gifted by Professor Satoshi Nagata, National Institutes of Biomedical Innovation, Health and Nutrition, Japan).

### Flow cytometry cell sorting

PBMCs were resuspended in FACS buffer at 2 × 10^7^ cells/ml. Cells were blocked with 4 μg/ml of human IgG (Octagam, Octapharma Ltd.) and stained with the antibody mix (Table S5) for 30 min at 4° celsius. Cells were then washed with PBS prior to labelling with Zombie UV (Biolegend) at 4° celsius for 15 min to assess viability. Stained cells were resuspended in FACS buffer at 1 × 10^7^ cells/ml. Prior to sorting, the cells were strained through a 30 μm cell strainer (CellTrics, Sysmex) to remove cell clumps. The cells were sorted on a BD FACSARIA II (BD Bioscience) with a 70 μm nozzle into the following subsets: Naïve B cells (CD19^+^IgD^+^CD27^−^), memory B cells (CD19^+^IgD^−^CD27^+^) and ABCs (CD19^+^CD11c^+^CD21^−^). A gating strategy for B‐cell subset sorting is shown in Figure S1b.

### Gene expression analysis––NanoString Technologies

For each B‐cell subset, 15 000 cells were sorted into RF10 (RPMI 1640 culture medium containing 10% FCS; both Sigma‐Aldrich). After sorting, the cells were pelleted and lysed in Buffer RLT (Qiagen) and stored at −80° celsius. Once sample collection was complete, the lysates were thawed on ice and analysed using the NanoString nCounter Human Immunology V2 Panel (NanoString Technologies) following the manufacturers' instructions.

The data analysis was performed using R (software version 3.5.3; The R Foundation) in association with the Bioconductor repository [20]. Gene expression was normalized by dividing raw counts by sample‐specific size factors that have been determined by applying the median of ratios method to the housekeeping genes [21]. A variance stabilizing transformation was applied to remove the dependence of the variance on the mean for the purpose of visualization and clustering. A sample‐ and probe‐level quality control step was conducted using the arrayQualityMetrics and NanoStringQCPro Bioconductor packages, respectively. Four samples were flagged as outliers and were thus excluded from further analysis. Present/absent calls were obtained for each gene in each sample, and only genes that were called ‘Present’ in all samples of at least one condition were retained. The DESeq2 Bioconductor package was used to compare the gene expression profiles between different B‐cell subsets and disease groups. DESeq2 tests for differential expression by fitting a negative binomial generalized linear model (GLM) for each gene and performing Wald tests for the significance of the GLM coefficients [22]. Genes with a Benjamini–Hochberg adjusted *p* value <0.05 and a fold change >1.5 were considered to be differentially expressed.

### B‐cell stimulation and cytokine secretion analysis

Sorted B cells were cultured in RF10 supplemented with 2 mM l‐glutamine, 100 U/ml penicillin and 100 μg/ml streptomycin (all Sigma‐Aldrich) at a density of 20 000 cells per well in 96‐well round‐bottom plates. B cells were stimulated with a combination of the TLR7 ligand, Imiquimod (1 μg/ml, InvivoGen), TLR9 agonists, ODN 2216–CpG A and ODN 2006–CpG B (both 1 μg/ml, InvivoGen), Poke Weed Mitogen (PWM, 5 μg/ml, Sigma‐Aldrich), anti‐CD40 antibody (10 μg/ml, clone HB14, mouse IgG1, Biolegend), human IL‐21 (0.05 μg/ml, Miltenyi Biotech), human IL‐4 (0.05μg/ml, Immunotools) and IFN‐γ (0.02 μg/ml, Peprotech) in a final volume of 200 μl. B cells were cultured for 5 days at 37° celsius with 5% CO_2_ and then supernatants were frozen at −80° celsius prior to further analysis by the Meso Scale Discovery (MSD) U‐PLEX Custom Biomarker (human) immunoassay Scal, assessing six cytokines (GM‐CSF, IL‐6, IL‐10, IL‐12p70, IL‐23 and TNF‐α).

### Statistical analysis

Graphs were generated using the GraphPad Prism (GraphPad Software Inc.) or R. Non‐parametric analyses of variance (Kruskal–Wallis test and Friedman test with Dunn's post hoc analyses), and the Mann–Whitney *U* tests were used for multiple‐group and two‐group independent comparisons, respectively. Pearson correlations were performed for bivariate comparisons. Significance was defined when *p* < 0.05.

## RESULTS

### ABCs have a distinct gene expression profile

**FIGURE 1:**
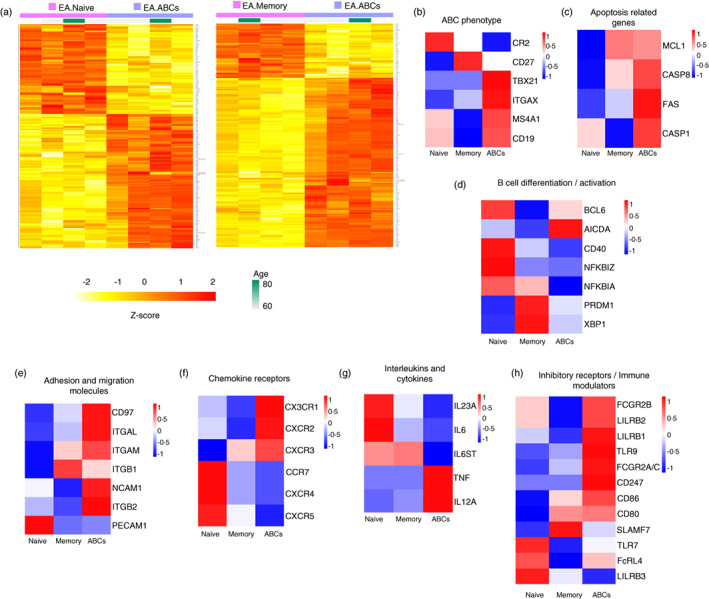
Age‐associated B cells (ABCs) from early rheumatoid arthritis (eRA) patients show a unique and distinct transcriptome profile compared to other B‐cell subsets. (a) Heatmap of the differentially expressed genes between ABCs and the other subsets of B cells: naïve B cells (left) and memory B cells (right). B‐cell subsets from eRA patients (*n* = 4) were sorted from peripheral blood mononuclear cell (PBMC) by flow cytometry. Cells were lysed, and the cell lysates were used for a NanoString nCounter® Immunology V2 Panel to assess gene expression. Gene expression profiles were compared between different B‐cell subsets using the DESeq2 R package. Normalized and variance stabilized gene expression intensities in log2 scale were used for clustering, and the corresponding *Z*‐scores were displayed with colours ranging from yellow to red as shown in the key. ABCs are shown in purple, and naïve and memory B cells are shown in pink. Each column represents a different eRA patient. Genes achieved statistical significance when the adjusted *p* value was <0.05 (FDR corrected), and the absolute fold change was >1.5. (b)–(h) Heatmaps showing the expression pattern of representative genes with relevant functions. For plotting, the *Z*‐score calculated from the mean log2 expression for each cell population was used. Red indicates higher expression, and blue indicates lower expression

In contrast to previous studies that have characterized ABCs in patients with estRA and healthy individuals, we studied ABCs in patients with DMARD‐naïve eRA. The donor demographics and clinical characteristics are shown in Table S1. B‐cell subsets from DMARD‐naïve eRA patients were flow cytometry cell sorted (see Figure S1b for gating strategy), and gene expression was assessed using a NanoString nCounter® Immunology V2 Panel. Differential gene expression analysis was performed after assessing data quality. Heatmaps were used to visualize clustering differences between cell types (Figure 1a). The ABCs were found to be a distinct subset of both naïve and memory B cells with a differential gene expression profile.

The ABCs had high levels of genes whose protein products were used to sort or have otherwise been described as ABC markers, such as *CD19*, *ITGAX* (CD11c), *TBX21* (T‐bet) and *MS4A1* (CD20), but low levels of *CR2* (CD21) (Figure 1b). ABCs also showed high expression of genes related to induction or regulation of apoptosis (Figure 1c). Genes involved in plasma cell differentiation (*PRDM1* BLIMP1] and *XBP1*) were more highly expressed in ABCs compared to naïve B cells, although ABCs had lower expression of *PRDM1* and *XBP1* compared to memory B cells (Figure 1d). Interestingly, *AICDA*, which is relevant for somatic hypermutation and class switching, was highly expressed in ABC when compared to naïve and memory B cells. Furthermore, analysis of migration and adhesion molecules showed high expression of the adhesion G protein‐coupled receptor family member, CD97, but not *PECAM1* (Figure 1e). The chemokine receptor profile of ABCs included high expression of inflammatory‐associated and RA synovium‐localization chemokine receptor genes, such as *CXCR3* and *CX3CR1* (fractalkine receptor), and low expression of chemokine receptor genes whose products mediate migration of leucocytes into and within lymphoid organs, such as *CXCR4* and *CXCR5* (Figure 1f). Moreover, ABCs had high expression of *TNF* and *IL12A* and low expression of *IL23A*, *IL6* and *IL6ST*, the gene encoding for the IL‐6 receptor beta subunit gp130 (Figure 1g). Additionally, ABCs showed high expression of inhibitory receptor‐encoding genes such as *FCGR2A/C*, *FCGR2B* and *LILRB1‐3*, as well as immunomodulatory molecules, including *TLR9*, *CD80* and *CD86* (Figure 1h). Compared to memory B cells, ABCs had higher expression of *FCRL4* but similar levels to those in naïve B cells.

### ABCs have a unique phenotypic profile

**FIGURE 2:**
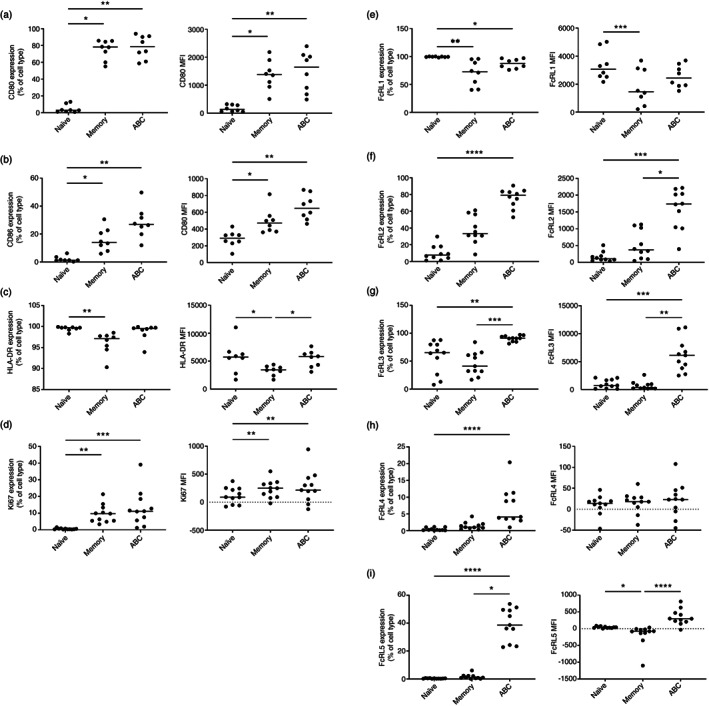
Age‐associated B cells (ABCs) are an activated, proliferating subset of B cells with high expression of co‐stimulatory molecules and HLA‐DR and elevated expression of the FcRL family members. Whole blood from early rheumatoid arthritis (eRA) patients was stained with an antibody panel for flow cytometry analysis. The percentage of positive cells for each marker in the B‐cell subsets is shown in the first panel (gated as outlined in Figure S1a) and the median fluorescence intensity (MFI) in the second panel. The horizontal line represents the median value. Statistical significance was assessed using the Friedman test with Dunn's multiple comparisons of each subset against the others; *p < 0.05; **p < 0.01; ***p < 0.001; ****p < 0.0001. CD80 (a), CD86 (b), HLA‐DR (c), Ki67 (d), FcRL1 (e), FcRL2 (f), FcRL3 (g), FcRL4 (h) and FcRL5 (i)

We next validated the expression of selected genes at the protein level using flow cytometry (Figures 2a, b and h and S3a–c). ABCs expressed high levels of co‐stimulatory molecules (CD80 and CD86) and T‐bet. In keeping with published literature, we demonstrated ABCs expressed high levels of the MHC class II molecule, HLA‐DR and the proliferative marker, Ki67 (Figure 2c,d), as well as expressing the activation marker, CD69 (Figure S3d). ABCs had a class‐switched immunoglobulin profile that more closely resembled memory B cells, with high frequencies of IgG and IgA and low frequencies of IgM‐ and IgD‐expressing cells compared to naïve B cells (Figure S3e–h).

While ABCs had a similar phenotype to memory B cells, there were key differences, mainly in FcRL family member expression (Figure 2e–i). FcRL1 expression was similar on ABCs and memory B cells but was lower compared to naïve B cells (Figure 2e). FcRL2, FcRL3 and FcRL5 were expressed at high levels in ABCs compared to both memory and naïve B cells (Figure 2f, g and i). Differing from the transcript data, expression of FcRL4 at the protein level showed no significant difference between ABCs and memory B cells, but there was a significantly higher frequency of ABCs expressing FcRL4 compared to naïve B cells (Figure 2h).

### The cytokine secretion profile following in vitro stimulation differs between B‐cell populations

**FIGURE 3:**
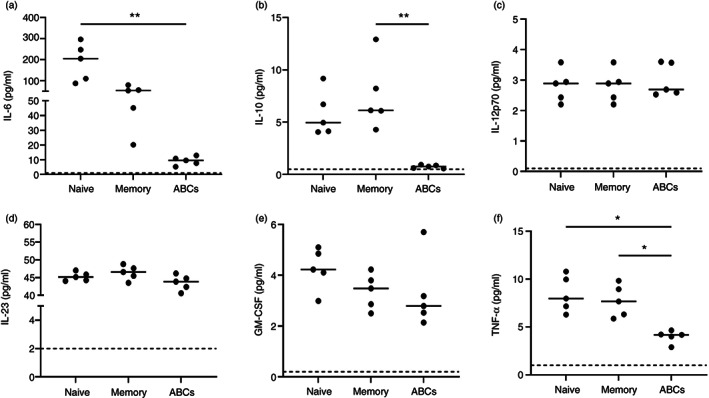
The cytokine secretion profile of age‐associated B cells (ABCs) is different from other B‐cell subsets. Sorted cells from established rheumatoid arthritis patients (*n* = 5) were incubated with a stimulation cocktail (Imiquimod, CpG A, CpG B, PWM, anti‐CD40, IL‐21, IL‐4 and IFN‐γ) for 5 days. The culture supernatants were harvested, and the concentration of each cytokine was detected using a U‐PLEX Custom Biomarker immunoassay from MSD. The dotted line indicates the unstimulated levels. Statistical significance was assessed using the Kruskal–Wallis test with Dunn's multiple comparisons of the two conditions in each B‐cell subset; *p < 0.05; **p < 0.01. IL‐6 (a), IL‐10 (b), IL‐12p70 (c), IL‐23 (d), GM‐CSF (e) and TNF‐α (f)

Expression of cytokine transcripts differed in ABCs compared to naïve and memory B cells (Figure 1g). Gene expression of IL‐10 and GM‐CSF did not pass QC due to low expression. However, as both are important B‐cell cytokines [23, 24], their protein secretion by the B‐cell subsets from estRA patients (the demographics and clinical characteristics of these donors are shown in Table S3) was assessed using an MSD immunoassay following stimulation. ABCs showed low secretion of IL‐6 and IL‐10 compared to naïve and memory B cells, respectively (Figure 3a,b). However, there was little or no difference in the secretion of IL‐12p70, IL‐23 and GM‐CSF between the different B‐cell subsets (Figure 3c–e). Interestingly, ABCs secreted low levels of TNF‐α compared to both naïve and memory B cells (Figure 3f).

### ABCs show distinct chemokine receptor and adhesion molecule expression

**FIGURE 4:**
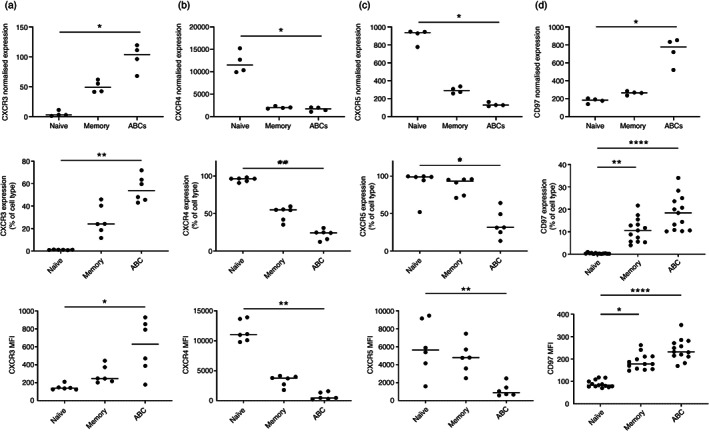
Age‐associated B cells (ABCs) show distinct patterns of chemokine receptor and adhesion molecule expression. The first‐row graphs show housekeeping gene‐normalized gene expression of the validated markers in early rheumatoid arthritis (eRA) patients (*n* = 4). For the validation, whole blood from eRA patients (*n* = 6) was stained with an antibody panel for flow cytometry analysis. The percentage of positive cells for each marker in the B‐cell subsets (gated as outlined in Figure S1a) is shown in the second row of panels and the median fluorescence intensity (MFI) in the third row. The horizontal line represents the median value. Statistical significance was assessed using the Friedman test with Dunn's multiple comparisons of each subset against the others; *p < 0.05; **p < 0.01; ****p < 0.0001. CXCR3 (a), CXCR4 (b), CXCR5 (c) and CD97 (d)

Gene expression analysis showed a unique pattern of chemokine receptors and adhesion molecules in ABCs (Figure 1e,f). Expression of CXCR3, CXCR4, CXCR5 and CD97 was all validated at the protein level using flow cytometry (Figure 4), suggesting ABCs may be more prone to migrate towards inflammatory sites rather than lymph nodes. Other chemokine receptors and adhesion molecules were not investigated at the protein level.

### ABCs are more abundant in SF, show a more activated and proliferative phenotype and their FcRL3, FcRL4 and FcRL5 expression differs from their PB counterparts

**FIGURE 5:**
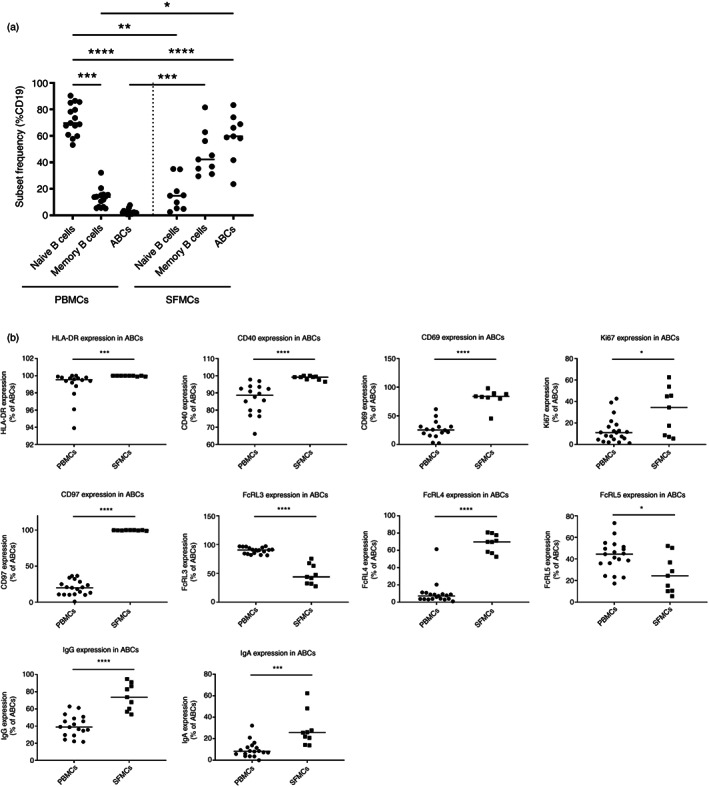
Age‐associated B cells (ABCs) are more abundant in synovial fluid (SF), show a more activated and proliferative phenotype, and their FcRL3, FcRL4 and FcRL5 expression differ from their peripheral blood (PB) counterparts. B‐cell subsets were detected by flow cytometry in the PB or SF of a cross‐sectional cohort of RA patients. The cell subsets were gated as outlined in Figure S1a. PB mononuclear cell (PBMC) (*n* = 19) and SF mononuclear cell (SFMC) (*n* = 9). (a) The frequency of each B‐cell subsets in PBMCs and SFMCs is shown as a percentage of total CD19^+^ B cells. (b) The percentage of positive cells for each marker in the ABC subset is shown. The horizontal lines represent the median value. Statistical significance was assessed using the Mann–Whitney *U* test; *p < 0.05; **p < 0.01; ***p < 0.001; ****p < 0.0001

As their expression profiles of chemokine receptors and adhesion molecules suggested ABCs may be able to migrate into inflammatory tissues and the joint synovium, we sought to investigate and characterize B cells in SF and PB from a cross‐sectional cohort of RA patients. In SF, a high proportion of B cells had an ABC‐like phenotype compared to PB (Figure 5a). Moreover, ABCs from SF showed differences in expression of certain markers compared to their PB counterparts (Figure 5b). SF ABCs had a higher proportion of cells expressing HLA‐DR, CD40, CD69, Ki‐67 and CD97. FcRL family member expression by SF ABCs also differed, with higher proportions of cells expressing FcRL4 and lower proportions of cells expressing FcRL3 and FcRL5 in SF compared to PB. In addition, SF B cells contained a higher proportion of cells expressing IgG and IgA compared to their PB counterparts.

### ABCs from eRA patients have a distinct transcriptional profile compared to control groups

Finally, after the characterization of ABCs in RA, we investigated whether ABCs from eRA patients phenotypically differ from those from estRA, early PsA (ePsA) patients and age‐matched HC. The demographics and clinical characteristics of these donors are shown in Table S1.

The proportion of ABCs, as well as naïve and memory B cells, in PB did not differ between the different disease groups and HCs (Figure S4a). Interestingly, female eRA patients had a higher frequency of circulating ABCs compared to male eRA patients (Figure S4b). This pattern was not seen in estRA, ePsA or HC (Figure S5a, S5c and S5e, respectively). In addition, there was no association of ABC frequencies with age in any of the groups (Figures S4c, S5b, s5d and S5f). Moreover, no differences were seen in expression of the phenotypic markers in ABCs from the different groups, with the exception of increased T‐bet expression in ABCs from HCs compared to estRA and ePsA (Figure S6g).

**FIGURE 6:**
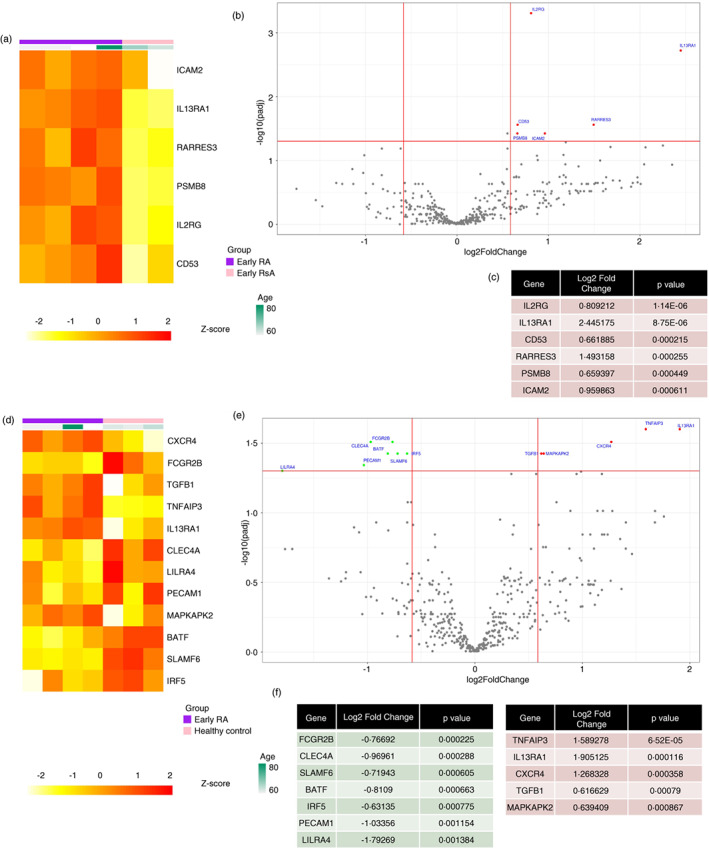
Age‐associated B cells (ABCs) from early rheumatoid arthritis (eRA) patients have a differential gene expression profile compared to control groups. Cell lysates from sorted ABCs from eRA, psoriatic arthritis (ePsA) and healthy controls (HCs) were loaded on to a NanoString nCounter® Immunology V2 Panel chip to assess gene expression. (a) and (d) Heatmap of the differentially expressed genes between ABCs from eRA patients (*n* = 4) in purple and ABCs from ePsA patients (*n* = 2) in pink (a) and between ABCs from eRA patients (*n* = 4) in purple and ABCs from HC (*n* = 3) in pink (d). Gene expression intensities were transformed to *Z*‐scores and are displayed as colours ranging from yellow to red as shown in the key. (b) and (e) Volcano plot showing the log2 fold change against the –log10 *p* value, genes plotted in red are upregulated and genes in green are downregulated in ABCs from eRA patients compared to ABCs from ePsA patients (b) and ABCs from HC (e). Genes achieved statistical significance when the adjusted p value was <0.05 (FDR corrected) and the absolute fold change was >1.5. (c) Table showing significantly upregulated genes, with their fold change and adjusted *p* value. (f) Table showing significantly downregulated genes (green) and upregulated genes (red), with their fold change and adjusted *p* value

Transcriptomic analysis of ABCs from eRA, ePsA and age‐matched HCs was conducted. The demographics and clinical characteristics of these donors are shown in Table S2. When assessed at the transcriptome level, the gene expression profile of ABCs from eRA differed to ABCs from ePsA and HCs (Figure 6). During the quality control analysis, two of the samples from the ePsA patients and one sample from a HC did not pass the quality control criteria and were therefore excluded, leaving only two ePsA ABC samples and three HC ABC samples. Heatmaps were used to visualize clustering differences between the eRA patients and the ePsA disease controls (Figure 6a) or the HC (Figure 6d). ABCs from eRA patients and ePsA patients cluster separately, as do ABCs from eRA patients and HC.

The genes identified as differentially expressed between eRA and ePsA patients were all upregulated in eRA and included subunits of interleukin receptors (*IL2RG* and *IL13RA1*), the retinoid acid receptor *RARRES3*, *CD53*, the adhesion molecule *ICAM2*, and *PSMB8*, which codes for a proteasome 20 S subunit (Figure 6b,c). Comparing ABCs from eRA patients with those from HC revealed downregulation in eRA of the transcription factors *BATF* and *IRF5*, as well as the adhesion molecule *PECAM1*, and Leucocyte Immunoglobulin‐Like Receptor (*LILRA4*) (Figure 6e,f). Upregulated genes in eRA included interleukin receptors, such as *IL13RA1*, the chemokine receptor *CXCR4*, TNF induced protein 3 (*TNFAIP3*), TGF‐beta 1 coding gene (*TGFB1*) and the kinase *MAPKAPK2*.

## DISCUSSION

This study has characterized ABCs in the blood and SF of DMARD‐naïve eRA patients. Our data support the idea, and corroborate published literature, that ABCs are a class‐switched memory B‐cell population with an activated, proliferative phenotype and T‐bet expression. However, our data also support the notion that the cell subset defined as ABCs are in fact a heterogeneous population [18, 25]. The best example to illustrate this heterogeneity is the expression of immunoglobulins by ABCs, as around half of these cells are positive for IgD, and around half of the cells are positive for the class‐switched immunoglobulin, IgG. This heterogeneity, combined with their high level of proliferation, may also support the view that the CD21^low^ CD11c^high^ phenotype may identify a differentiation stage following on from activation rather than a stable, fully differentiated B‐cell subset. This is also in agreement with the increased levels of these cells in conditions involving a high degree of activation of the immune system, such as malaria, viral infection and active SLE [3, 26, 27].

Interestingly, we have shown that PB ABCs have an elevated expression of the FcRL family members, FcRL2‐5, similar to that reported for ABC‐like cells from granulomatous lung diseases [28]. Expression of FcRL3, FcRL4 and FcRL5 on ABC‐like cells from SLE and malaria infection has also been reported [14, 26, 29, 30]. FcRL3 is of interest as a likely mediator of genetic risk in RA. We have shown its transcript to be subject to both an expression and a methylation quantitative trait locus in CD4^+^ T cells and B cells [31, 32]. In addition, our previous work demonstrates that FcRL4^+^ B cells are a pro‐inflammatory B‐cell subset found enriched in RA inflamed joints [11, 12]. In this study, we found differences in FcRL family member expression between SF ABCs and those from PB: FcRL3 and FcRL5 are decreased in SF ABCs, but FcRL4 is increased, in agreement with the observation that SF CD21^−/low^ B cells from RA SF have increased FcRL4 expression compared to their PB counterparts [9]. Interestingly, both FcRL3 and FcRL4 are proposed to be IgA‐binding receptors [33, 34] with the ability to modulate B‐cell responses; both FcRL3 and FcRL4 have been reported to act as molecular switches, enhancing TLR9‐mediated responses but inhibiting BCR signalling [35, 36, 37].

We demonstrate that following in vitro activation, ABCs from estRA secrete lower levels of IL‐6, IL‐10 and TNF‐α but similar levels of other cytokines, such as IL‐23, IL‐12 and GM‐CSF, compared to naïve and memory B cells. This cytokine profile, taken together with their high expression of the MHC class II antigen presentation molecule, HLA‐DR, as well as high expression of co‐stimulatory molecules, CD80 and CD86, may suggest a possible role in antigen presentation to T cells. Interestingly, ABCs have low expression of *IL6ST*, the gene that encodes the IL‐6 receptor subunit, gp130, which suggests ABCs may have reduced responses to gp130‐dependent cytokine pathways, such as IL‐6.

We also demonstrated that ABCs have a distinct chemokine receptor profile, with high expression of CXCR3 and *CXCR2*, both encoding receptors, which recruit cells to inflamed tissues, and *CX3CR1*, which has been linked to the migration of cells to the RA synovium [38], and low expression of CXCR4, CXCR5 and *CCR7*, which mediate recruitment to lymph nodes. Interestingly, another study demonstrated that T‐bet^+^ B cells with high expression of CXCR3, akin to the ABCs reported here, were increased in cerebrospinal fluid from patients with MS and had a higher migration capacity, as assessed in transwell assays [39]. Taken together, these data support the potential for ABCs to migrate into inflamed synovial tissue. This hypothesis is further supported by the higher percentages of ABC‐like cells in the SF of patients with RA. However, another hypothesis based on recent findings [40, 41] is that SF ABCs represent a tissue‐resident rather than a re‐circulating B‐cell subset, meaning these cells develop in the joint rather than migrate in. Further investigation of this hypothesis is required to understand the developmental pathway of these cells.

The frequencies of ABCs in the SF of patients with RA were much higher than the frequencies found in the blood. It was not possible to obtain matched blood and SF from the same patients, therefore, a cross‐sectional study had to be conducted. However, these results are in line with other previously published studies in RA [9, 11, 12]. In addition, a recent single‐cell study of RA synovium has identified an enrichment of ABC‐like cells (CD20^+^T‐bet^+^CD11c^+^ B cells) compared with osteoarthritis synovium [10]. Of interest, ABC‐like cells have also been reported to be enriched in bronchoalveolar lavage from pulmonary sarcoidosis patients compared to PB [28]. Even though ABCs are found in much higher frequencies in SF than in blood, further research is needed to understand their origin. It is still unknown if ABCs migrate from the blood into the tissue or if B cells from the blood infiltrate the inflammatory tissue and, once there, they are skewed towards an ABC phenotype.

In contrast to a recent study [42], we did not find that the frequency of ABCs was increased in either eRA or estRA compared to age‐matched HC. This discrepancy may be explained by the difference in the definition of ABCs: We defined ABCs as CD21^−^CD11c^+^ B cells, whereas Bao et al. defined them as T‐bet^+^CD11c^+^ B cells. We found that CD21^−^CD11c^+^ ABCs have heterogeneous expression of T‐bet, and therefore our two studies are not investigating the exact same cell population. Interestingly, at the transcriptome level, ABCs from DMARD‐naïve eRA differ from those in ePsA and HC, with a larger difference seen between eRA and HC. For example, the gene *IL13RA1*, a subunit of the functional receptor for IL‐13 [43], is upregulated in ABCs from eRA compared to ePsA and HC. IL‐13 is increased in SF from eRA compared to estRA and other inflammatory arthritides [44], which may highlight a role for IL‐13 signalling in eRA ABCs. A major consideration regarding these findings is the small sample size used. Only four individuals per group were recruited due to the need for recruiting age‐matched HC, and the isolation of sufficient numbers of B‐cell subsets for analysis, and due to the loss of two PsA and one HC due to QC failure, it is difficult to draw strong conclusions from the data shown. In addition, due to the high heterogeneity present in RA, large sample sizes are often needed to overcome individual variability; therefore, further validation of these findings is required.

In conclusion, ABCs are an activated B‐cell population which could potentially migrate into inflammatory sites and promote disease pathogenesis by acting as antigen‐presenting cells. The ABCs from RA differ from their counterparts in other disease states as well as health, suggesting that ABCs may contribute to the immune dysregulation seen in RA. However, further functional studies are still needed to fully unravel the role of ABCs in health and disease.

## AUTHOR CONTRIBUTIONS

Amy E. Anderson, Arthur G. Pratt, Andrew L. Mellor, Dagmar Scheel‐Toellner and John D. Isaacs conceptualized the study. Amy E. Anderson, Dagmar Scheel‐Toellner, Gemma Vidal‐Pedrola and John D. Isaacs designed the experiments. Gemma Vidal‐Pedrola performed experiments. Gemma Vidal‐Pedrola and Najib Naamane analysed the data and Amy E. Anderson, Arthur G. Pratt, Dagmar Scheel‐Toellner, Gemma Vidal‐Pedrola, James A. Cameron and John D. Isaacs interpreted the data. Amy E. Anderson and Gemma Vidal‐Pedrola wrote the initial draft of the manuscript. All authors revised and approved the submitted manuscript version.

## FUNDING INFORMATION

This work was funded by the Research into Inflammatory Arthritis Centre Versus Arthritis (RACE) (grant number 22072) and supported by the J.G.W. Patterson Foundation, the EU/EFPIA Innovative Medicines Initiative 2 Joint Undertaking RTCure (grant number 777357) and the National Institute for Health and Care Research (NIHR) Newcastle Biomedical Research Centre (BRC) at the Newcastle Hospitals NHS Foundation Trust and Newcastle University, UK. John D. Isaacs is a NIHR Senior Investigator. The views expressed are those of the author(s) and not necessarily those of the National Institute for Health Research or the Department of Health and Social Care.

## CONFLICT OF INTEREST

The authors declare no competing commercial or financial interests in relation to the work described.

## Supporting information


**Appendix S1.** Supporting Information.

## Data Availability

The data that support the findings of this study are available on request from the corresponding author. The data are not publicly available due to privacy or ethical restrictions.
