# Supplementary material for: Characterization of age‐associated B cells in early drug‐naïve rheumatoid arthritis patients
Source: Immunology. 2022 Nov 10;168(4):640–53. doi: 10.1111/imm.13598 (PMC11495260; doi:10.1111/imm.13598)
Supplement: Supplementary file 1 — Appendix S1. Supporting Information. [file IMM-168-640-s001.docx]

**Characterisation of age-associated B cells in early drug-naïve rheumatoid arthritis patients**

**Supplementary material**

**Supplementary tables**

|  | **eRA patients**  (n=50) | **estRA patients**  (n=12) | **PsA patients**  (n=17) | **HC**  (n=16) | **p value (inflammatory diagnosis*)** | **p value (all diagnosis)** |
| --- | --- | --- | --- | --- | --- | --- |
| Age (years) | 68  (21 - 89) | 62.5  (31 - 84) | 47.8  (22-79) | 61  (25 - 73) | 0.006^#^ | 0.0026^#^ |
| Female, % | 68 | 75 | 47 | 61.5 | 0.2116^+^ | 0.35^+^ |
| CRP (g/L) | 9  (4-127) | 8.5  (4-78) | 7  (4-167) | - | 0.5949 | - |
| DAS28 | 4.47  (1.33 – 8.53) | 1.38  (0.68 – 3.74) | - | - | 0.0001^ǂ^ | - |
| Seropositive – anti-CCP+ or RF+, % | 68 | 92 | 18 | - | <0.0001^+^ | - |

**Supplementary Table 1.  Demographic and clinical characteristics for the cohorts used for B-cell phenotypic characterisation.** Demographical data for the disease cohorts: eRA patients (n=50); estRA patients (n=12); PsA patients (n=17); and the HC cohort (n=16) used to determine the frequency of ABCs in blood. Except where indicated, median and range are given. Anti- CCP = anti-cyclic citrullinated peptide antibody, RF = rheumatoid factor. * Comparison of eRA, estRA and PsA patients. # Kruskal-Wallis test with Dunn’s multiple comparisons. + Chi-square test. ǂ Mann-Whitney test. The groups were matched for sex and the eRA, estRA and PsA groups were all matched for inflammation, as measured by CRP. The PsA patient group were younger than the other groups and had a lower percentage of seropositive patients compared to the RA groups. The eRA group had a higher disease activity, measured by DAS28, than the estRA group.

|  | **eRA patients**  (n=4) | **ePsA patients**  (n=4) | **HC**  (n=4) | **p value (inflammatory diagnosis*)** | **p value (all diagnosis)** |
| --- | --- | --- | --- | --- | --- |
| Age (years) | 70  (60 - 80) | 70  (63 - 79) | 63  (61 - 73) | >0.9^#^ | >0.9^#^ |
| Females, % | 50 | 50 | 50 | >0.9^+^ | >0.9^+^ |
| CRP (g/L) | 12  (4-23) | 8.5  (2-17) | - | 0.4571 | - |
| DAS28 | 5.74  (4.65 - 6.37) | - | - | - | - |
| Seropositive – anti-CCP+ and RF+, % | 100 | 0 | - | 0.03^ǂ^ | - |

**Supplementary Table 2. Demographic and clinical characteristics for the cohorts used for NanoString gene expression analysis.** Flow cytometry cell sorting was performed on the disease cohorts: eRA patients, (n = 4) and ePsA patients, (n = 4) and the HC cohort (n = 4) to assess gene expression using NanoString Technologies. Except where indicated, median and range are given. * Comparison of eRA and ePsA patients. # Kruskal-Wallis test with Dunn’s multiple comparisons. + Chi-square test. ǂ Fisher's exact test. There was no difference in age and sex between the groups, and while seropositivity was (as expected) higher for the eRA group compared to the ePsA, there was no difference in inflammation.

|  | **estRA patients**  (n=5) |
| --- | --- |
| Age (years) | 67  (31 - 72) |
| Females, % | 80 |
| CRP (g/L) | 7  (4-12) |
| DAS28 | 1.38*  (1.38-1.38) |
| Seropositive – anti-CCP+ and RF+, % | 100 |
| Treatment:  MTX, %  HCQ, % | 100  20 |

**Supplementary Table 3. Demographic and clinical characteristics for the estRA cohort used for cytokine production analysis.** Except where indicated, median and range are given. * DAS28 only available for 2/5 patients. HCQ, hydroxychloroquine; MTX, methotrexate.

| **Surface marker** | **Fluorophore** | **Clone** | **Dilution factor** | **Company** |
| --- | --- | --- | --- | --- |
| **CD3** | PE-CF594 | UCHT1 | 1:200 | BD Biosciences, CA, USA |
| **CD33** | PE-CF594 | WM53 | 1:33 | BD Biosciences, CA, USA |
| **CD19** | BUV395 | SJ25C1 | 1:100 | BD Biosciences, CA, USA |
| **CD20** | BV510 | 2H7 | 1:200 | Biolegend, CA, USA |
| **CD11c** | APC | S-HCL-3 | 1:200 | BD Biosciences, CA, USA |
| **CD11c** | BV421 | B-LY6 | 1:20 | BD Biosciences, CA, USA |
| **CD21** | PE | BU32 | 1:200 | Biolegend, CA, USA |
| **CD21** | PE-Cy7 | BU32 | 1:50 | Biolegend, CA, USA |
| **CD27** | BV650 | O323 | 1:200 | Biolegend, CA, USA |
| **IgD** | AF700 | IA6-2 | 1:100 | Biolegend, CA, USA |
| **CD80** | BB515 | L307.4 | 1:100 | BD Biosciences, CA, USA |
| **CD86** | BV711 | IT2.2 | 1:200 | Biolegend, CA, USA |
| **CD69** | APC-Vio770 | FN-50 | 1:50 | Miltenyi Biotech, Germany |
| **HLA-DR** | PerCP | L203 | 1:50 | RnD Systems, MN, USA |
| **CD40** | BV421 | 5C3 | 1:50 | Biolegend, CA, USA |
| **T-bet** | BV421 | O4-46 | 1:10 | BD Biosciences, CA, USA |
| **Ki67** | BV711 | Ki-67 | 1:10 | Biolegend, CA, USA |
| **FcRL3** | BB515 | H5 | 1:200 | BD Biosciences, CA, USA |
| **FcRL4** | PE | 413D12 | 1:100 | Biolegend, CA, USA |
| **FcRL5** | APC | 509f6 | 1:50 | Biolegend, CA, USA |
| **FcRL1** | APC | REA440 | 1:100 | Miltenyi Biotech, Germany |
| **FcRL2** | PE | B24 | 1:100 | Gift from Prof. Nagata, Japan |
| **IgM** | BB515 | G20-127 | 1:20 | BD Biosciences, CA, USA |
| **IgG** | BV786 | G18-145 | 1:50 | BD Biosciences, CA, USA |
| **IgA** | PE | IS11-8E10 | 1:50 | Miltenyi Biotech, Germany |
| **CXCR3** | AF488 | 1C6 | 1:50 | BD Biosciences, CA, USA |
| **CXCR4** | APC | REA649 | 1:100 | Miltenyi Biotech, Germany |
| **CXCR5** | BV510 | J252D4 | 1:100 | Biolegend, CA, USA |
| **CD95/FAS** | PE | REA738 | 1:100 | Miltenyi Biotech, Germany |
| **CD97** | FITC | VIM3b | 1:100 | Biolegend, CA, USA |

**Supplementary Table 4. Fluorophore labelled antibodies used for phenotyping.**

| **Surface marker** | **Fluorophore** | **Clone** | **Dilution factor** | **Company** |
| --- | --- | --- | --- | --- |
| **CD3** | PE-CF594 | UCHT1 | 1:12.5 | BD Biosciences, CA, USA |
| **CD33** | PE-CF594 | WM53 | 1:12.5 | BD Biosciences, CA, USA |
| **CD19** | BUV395 | SJ25C1 | 1:10 | BD Biosciences, CA, USA |
| **CD20** | BV510 | 2H7 | 1:12.5 | Biolegend, CA, USA |
| **CD11c** | APC | S-HCL-3 | 1:10 | BD Biosciences, CA, USA |
| **CD21** | PE | BU32 | 1:16.5 | Biolegend, CA, USA |
| **CD5** | PE-Cy7 | UCHT2 | 1:12.5 | eBioscience, CA, USA |
| **CD27** | FITC | M-T271 | 1:12.5 | BD Biosciences, CA, USA |
| **IgD** | AF700 | IA6-2 | 1:16.5 | Biolegend, CA, USA |

**Supplementary Table 5. Fluorophore labelled antibodies used for sorting of B cell subsets.**

**Supplementary Figures**

**
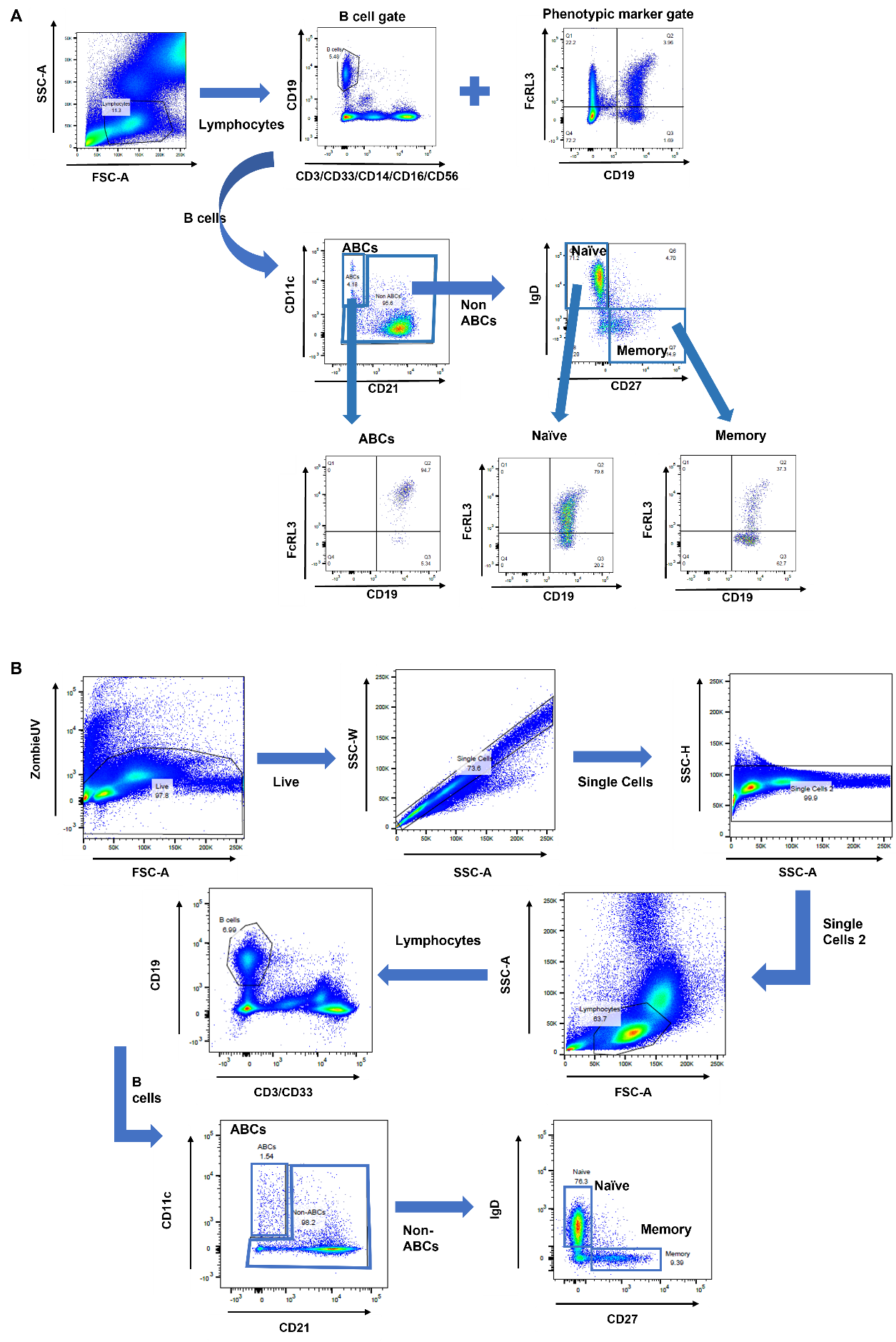
**

**Figure S.1. Gating strategy used for the phenotypic markers (A) and for the gating strategy for sorting each B-cell subset for gene expression analysis (B). A.** The gating strategy for determination of FcRL3 expression in B-cell subsets is shown as an example. A similar strategy was used for all the other phenotype markers. Doublets (SSC-W v SSC-A) were excluded (not shown). Lymphocytes were selected based on their forward and sideward scatter signal, B-cells were identified as CD19^+^ and negative for CD3, CD33, CD14, CD16 and CD56. Among the selected B-cells, the four B-cell subsets were defined. From the lymphocyte gate, the phenotypic marker against CD19 expression is gated and the phenotypic marker gate is copied in each B-cell subset, allowing determination of expression of a set of markers in each subset. The phenotypic marker’s gate is set on the expression of that marker in the CD19^-^ fraction of cells. If the CD19^-^ population also expresses the phenotypic marker, for example with FcRL3, a FMO strategy was used to set the gate ensuring ≤ 0.5% of events for the marker of interest were in the positive gate. **B.** The gating strategy used to sort the four B-cell subsets for gene expression analysis. After excluding dead cells and doublets (using two different gates), lymphocytes were gated using SSC-A vs FSC-A. From the lymphocyte gate, B-cells were gated using CD19 vs CD3/CD33 (to remove T-cells and myeloid cells). From the B-cell gate, the ABCs were gated using CD11c vs CD21 with ABC identified as CD11c^+^CD21^-^. From the remaining cells (non-ABCs) naïve (IgD^+^CD27^-^) and memory (IgD^-^CD27^+^) B-cells were gated. Gates were set based on an initial FMO ensuring ≤ 0.5% of events for the marker of interest were in the positive gate, and every time that a donor was recruited the gates were checked to ensure that the populations were inside the gate.


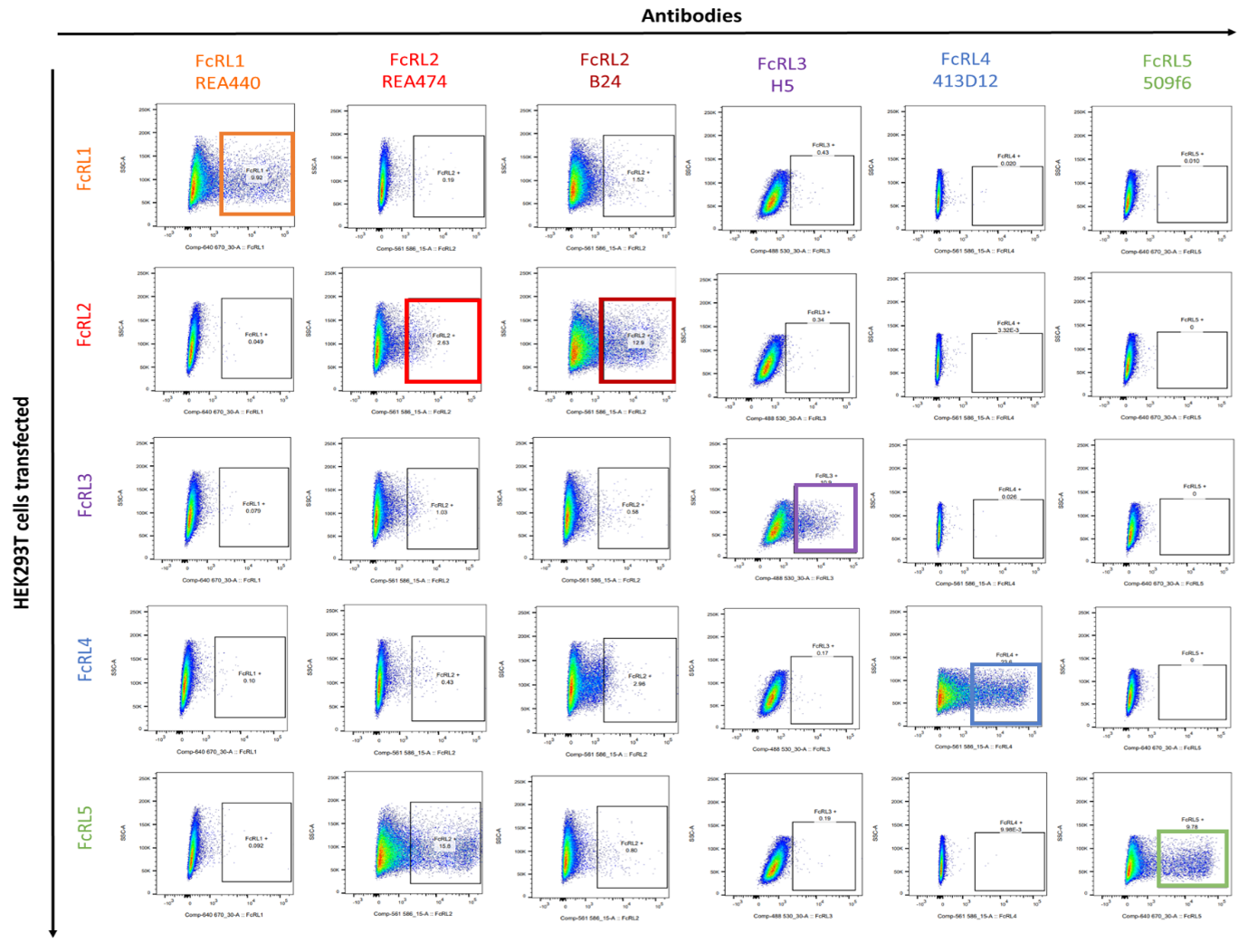


**Figure S.2. Anti-FcRL antibody specificity was tested using transfected HEK 293T-cells expressing a single FcRL.** HEK 293T-cells were transiently transfected with one of the FCRL expression plasmids, and 24 hours after transfection, the cells were stained individually with each fluorophore-conjugated monoclonal antibody and analysed by flow cytometry. Doublets and dead cells were excluded and cells were selected based on their forward and sideward scatter signal (data not shown). The expression of each FcRL was gated on the resulting alive cell population. An isotype control was used to set each FcRL gate, ensuring ≤ 0.5% of events for the marker of interest were in the positive gate. Each of the transfected cell lines expressing just one FcRL family member is presented in separate rows and each anti-FcRL antibody clone used to stain the transfected cells is presented in each column. Representative of two independent experiments.

**
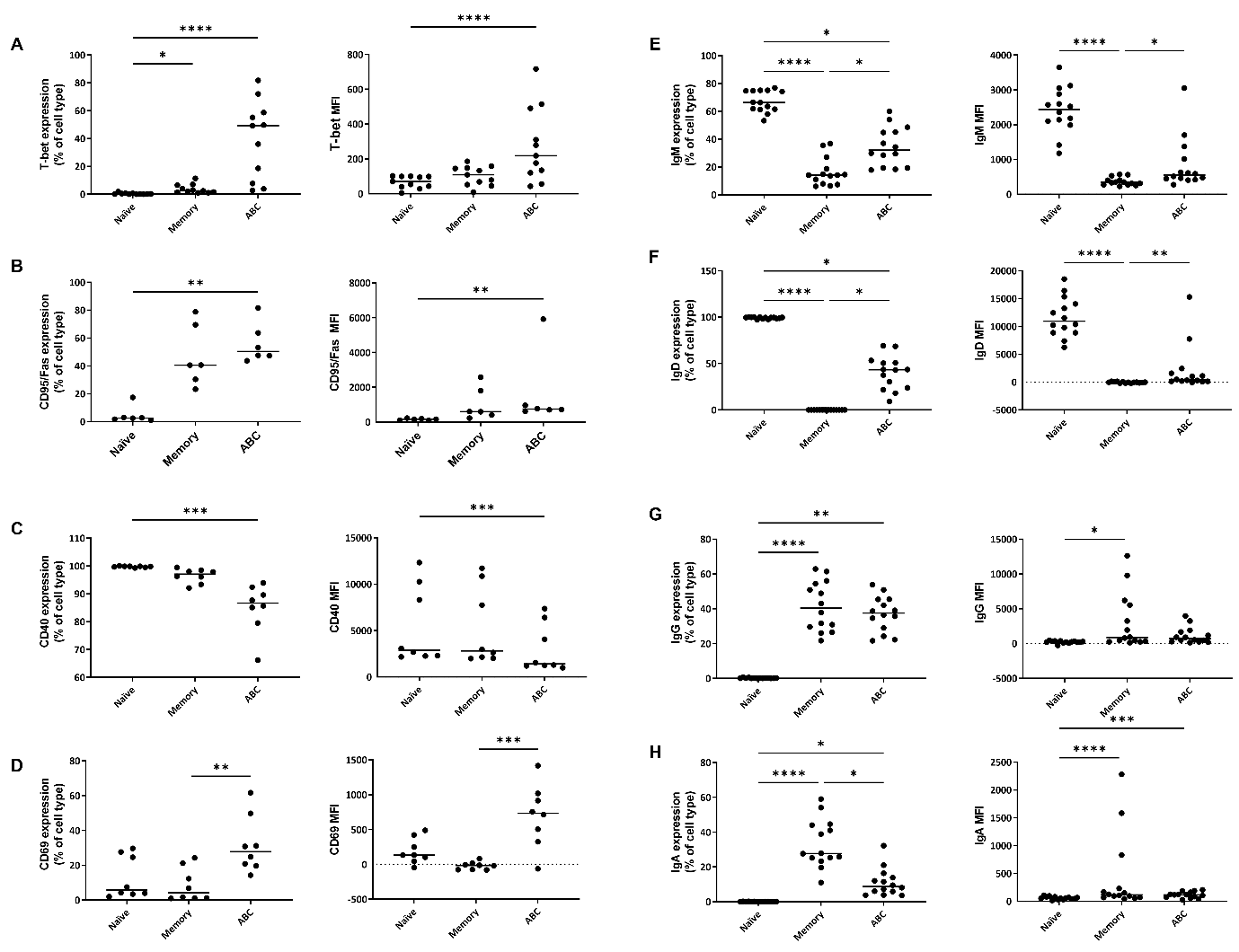
**

**Figure S.3. Additional phenotypic markers expression analysis.** Whole blood from eRA patients was stained with an antibody panel for flow cytometry analysis. For the immunoglobulin markers IgM, IgD, IgG, and IgA, PBMCs were isolated using Lymphoprep and were stained with the required flow cytometry antibodies. The percentage of positive cells for each marker in the B-cell subsets is shown in the first panel (gated as outlined in Supplementary Figure S.1.A) and the median fluorescence intensity (MFI) in the second panel. The horizontal line represents the median value. Statistical significance was assessed using a Friedman test with Dunn’s multiple comparisons of each subset against the others; * p < 0.05, **p < 0.01, *** p < 0.001, **** p < 0.0001. T-bet (**A**), CD95/Fas (**B**), CD40 (**C**), CD69 (**D**), IgM (**E**), IgD (**F**), IgG (**G**) and IgA (**H**).


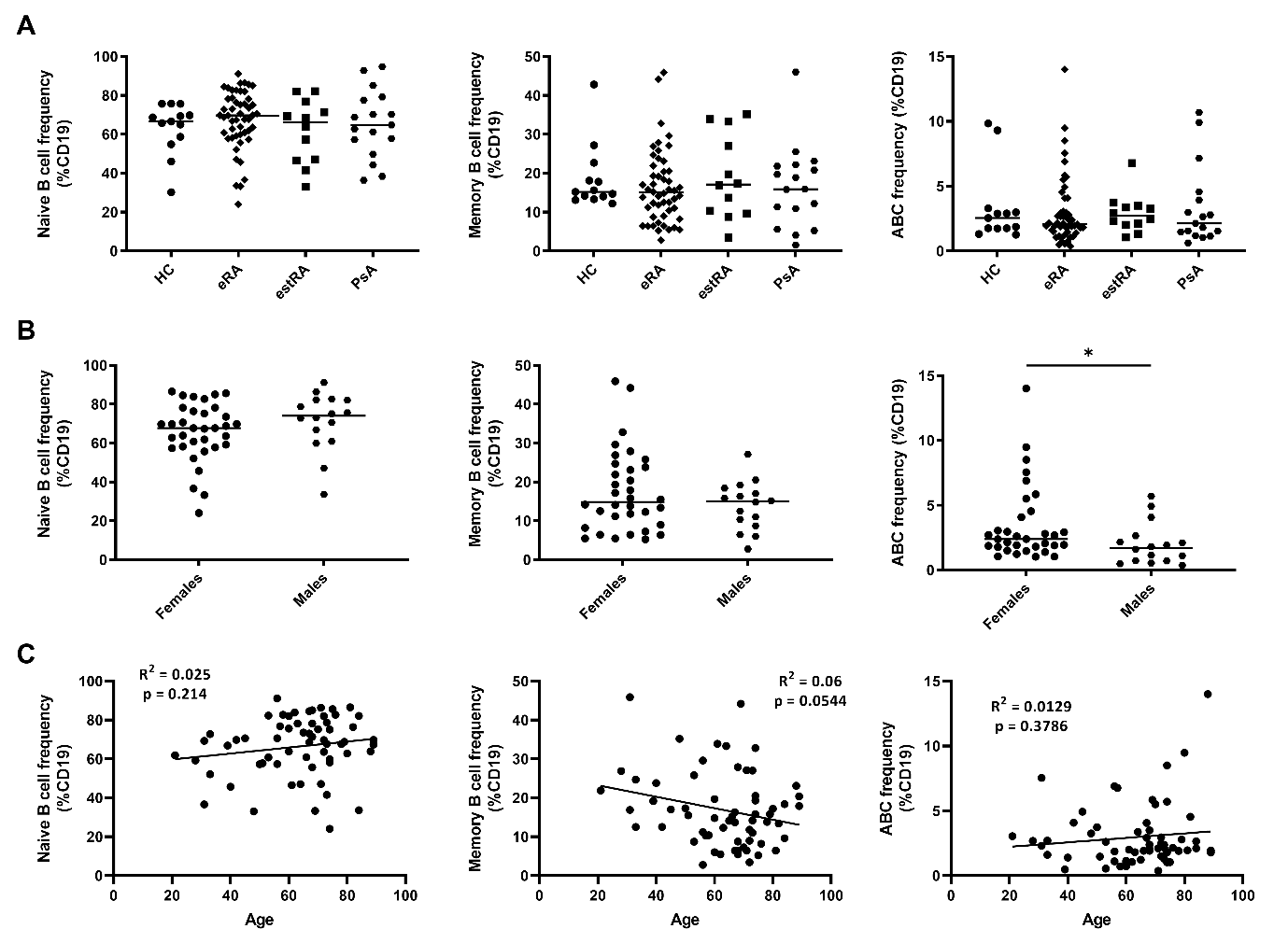


**Figure S.4. Percentages of each B-cell subsets in the different disease groups and the HC used for the phenotypic characterisation of ABCs.** B-cell subsets were detected by flow cytometry using whole blood staining (gated as outlined in Supplementary Figure S.1.A). The frequency of each B-cell subsets is shown as a percentage of total CD19+ B-cells. **A.** Naïve B-cells, memory B-cells and ABCs frequency in HC older than 50 years old (HC, n=13), eRA patients (eRA, n=50), estRA patients (estRA, n=12) and PsA patients (PsA, n=17). The median value is represented by the horizontal line for each group. No statistically significance differences were found using a Kruskal-Wallis test. **B.** B-cell subsets frequency in eRA patients separated by sex. Females (n=34) and males (n=16). The median value is represented by the horizontal line for each group. Statistical significance was assessed using a Mann- Whitney U test; * p < 0.05. **C.** Correlation of the B-cell subset frequency with the age in eRA patients, n=50. Statistical significance was assessed using a Pearson R test.


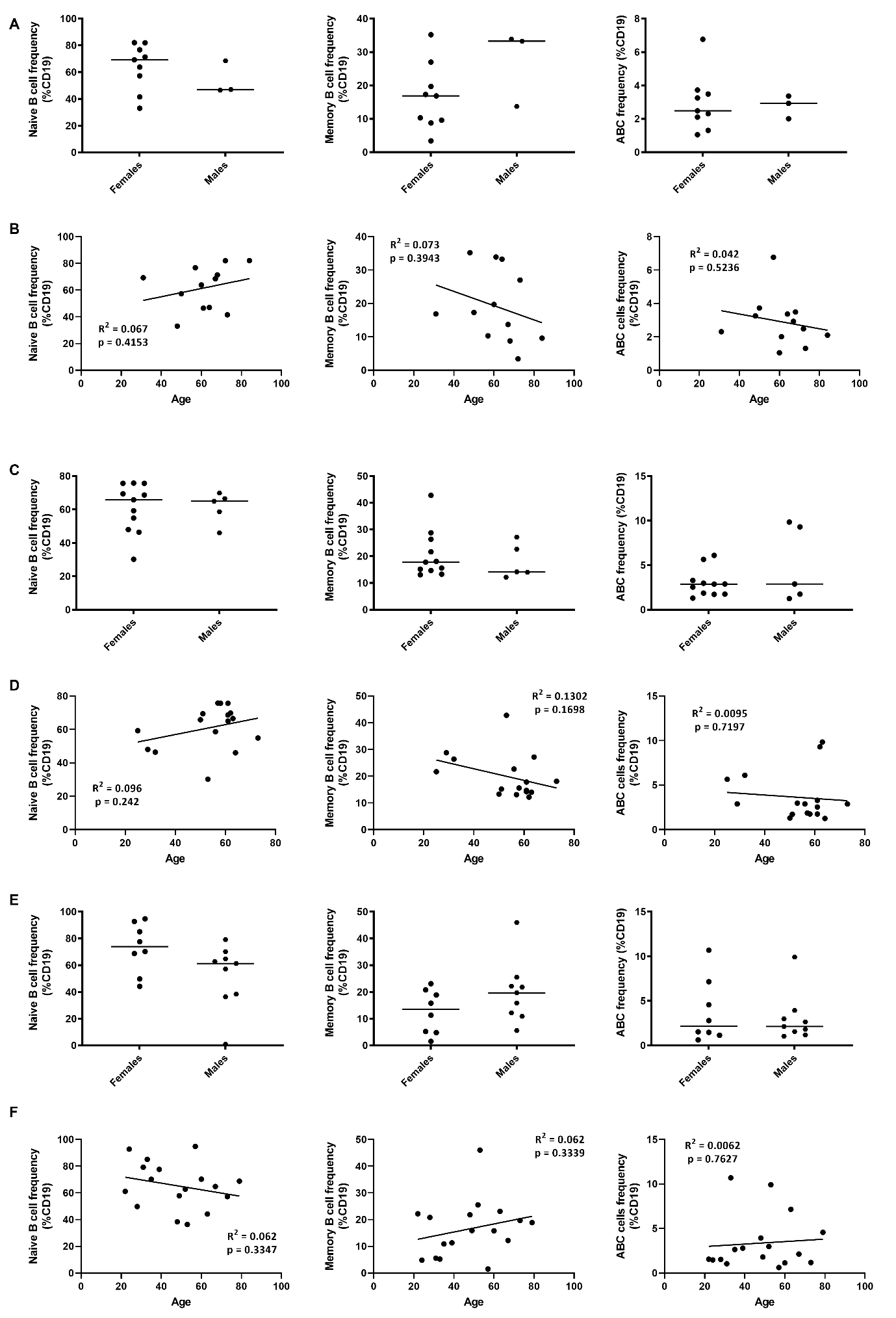


**Figure S.5. B-cell subsets frequency in patients with estRA, PsA, and age-matched HC.** B-cell subsets were detected by flow cytometry using whole blood staining (gated as outlined in Supplementary Figure S.1.A). The frequency of each B-cell subsets is shown as a percentage of total CD19+ B-cells. B-cell subsets frequency in females and males in (**A**) estRA patients (females (n=9) and males (n=3)), (**C**) early PsA patients (females (n=8) and males (n=9)), and (**E**) HC (females (n=11) and males (n=5)). The median value is represented by the horizontal line for each group. Statistical significance was assessed using a Mann- Whitney U test. Correlation of the B-cell subset frequency with the age in (**B**) estRA patients (n=12), (**D**) early PsA patients (n=17), and (**F**) HC (n=16). Statistical significance was assessed using a Pearson R test.


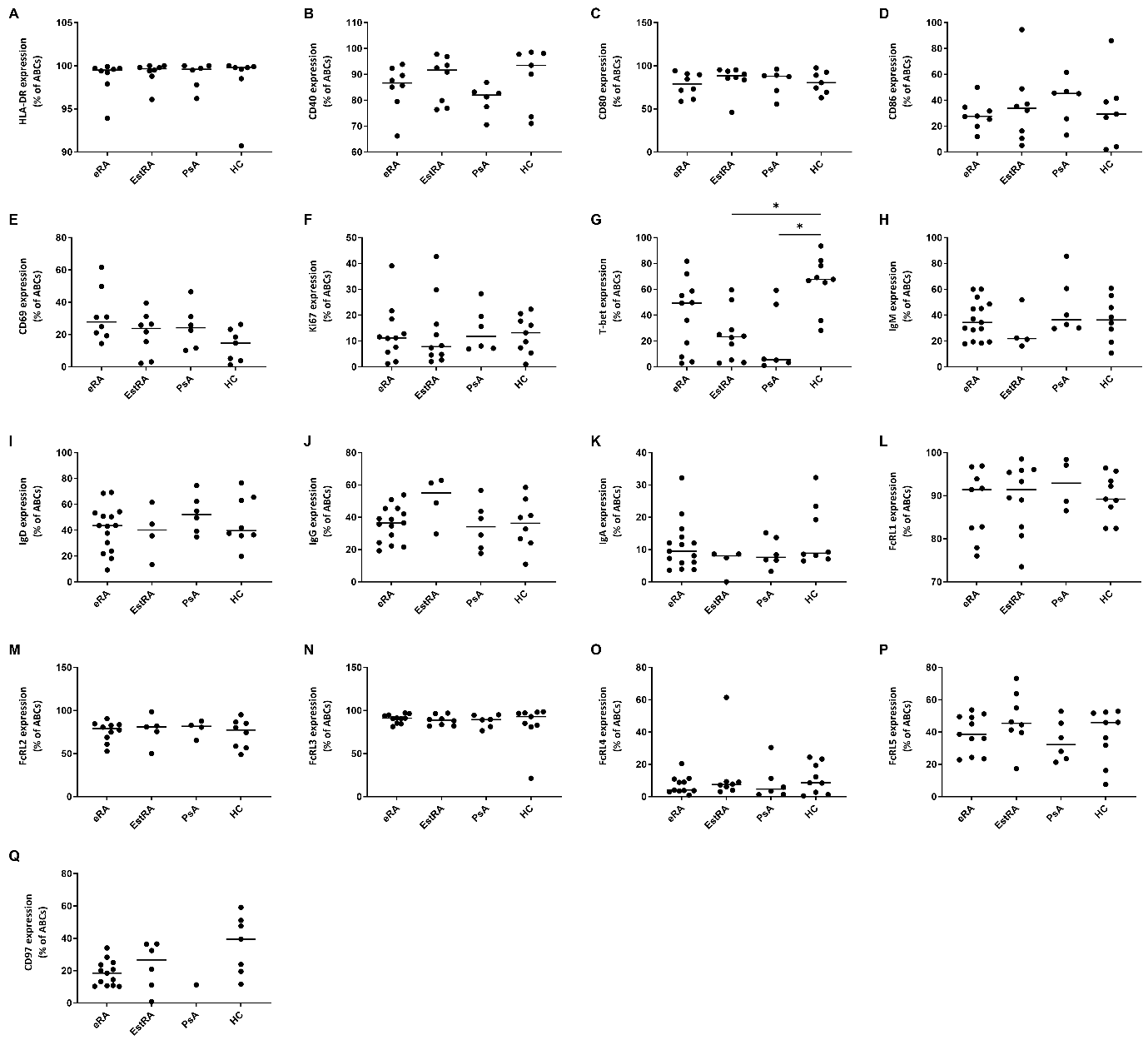


**Figure S.6. Expression of markers of interest in ABCs from different disease cohorts and HCs.** Disease controls and HC donors were recruited and whole blood was stained for different flow cytometry panels (gated as outlined in Supplementary Figure S.1.A). The percentage of cells positive for HLA-DR (**A**), CD40 (**B**), CD80 (**C**), CD86 (**D**), CD69 (**E**), Ki67 (**F**), T-bet (**G**), IgM (**H**), IgD (**I**), IgG (**J**), IgA (**K**), FcRL1 (**L**), FcRL2 (**M**), FcRL3 (**N**), FcRL4 (**O**), FcRL5 (**P**), and CD97 (**Q**) in the ABC subset in eRA patients, estRA patients, PsA disease controls and age-matched HC. The horizontal line represents the median. Statistical significance was determined using a Kruskal-Wallis test with Dunn’s multiple comparisons of each group against the others; * p < 0.05.
